# QuickStats

**Published:** 2015-02-06

**Authors:** FROM THE NATIONAL CENTER FOR HEALTH STATISTICS

**Figure:**
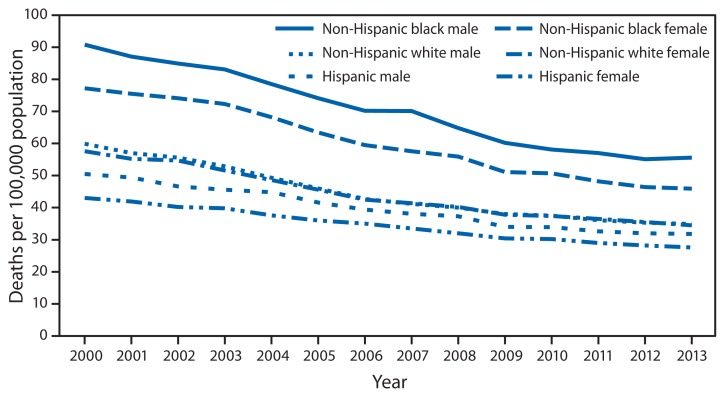
Age-Adjusted Death Rate* for Stroke,^†^ by Hispanic Ethnicity, Race for Non-Hispanic Population, and Sex — United States, 2000–2013 * Per 100,000 standard population. ^†^ As underlying cause of death, stroke is coded as I60–I69 in the *International Classification of Diseases, 10th Revision*.

During 2000–2013, age-adjusted death rates for stroke for all racial/ethnic groups decreased steadily. Non-Hispanic white males had the largest decline (41.7%), and Hispanic females had the smallest (35.8%). Throughout the period, the rate for non-Hispanic black was the highest among the racial/ethnic groups examined, followed by non-Hispanic white and Hispanic populations. The rate for males was higher than that for females in each racial/ethnic group.

**Source:** National Vital Statistics System. Mortality public use data files, 2000–2013. Available at http://www.cdc.gov/nchs/data_access/vitalstatsonline.htm.

**Reported by:** Jiaquan Xu, MD, jax4@cdc.gov, 301-458-4086.

